# Risk Transmission of Hg/MeHg from Aquatic Environments to Dietary Exposure Through Aquatic Products: Network-Based Pathway Identification and a Framework Toward Probabilistic Early Warning

**DOI:** 10.3390/foods15213879

**Published:** 2026-10-29

**Authors:** Li Wang, Yuanyuan Zhang, Xiaoyu Cui, Zhiyao Zhao, Jiabin Yu

**Affiliations:** School of Computer and Artificial Intelligence, Beijing Technology and Business University, Beijing 100048, China

**Keywords:** aquatic products, mercury, methylmercury, food web, network analysis, Bayesian networks, dietary exposure, probabilistic early warning

## Abstract

Mercury (Hg) and methylmercury (MeHg) can be transferred from aquatic environments through food webs to edible fishery products and ultimately contribute to human dietary exposure. This structured narrative review synthesizes 99 sources to examine the continuous risk-transmission process from environmental Hg/MeHg dynamics to dietary exposure and to evaluate the roles of network analysis, Bayesian networks (BNs), and Monte Carlo simulation in related risk assessment. Three main findings emerge. First, concentrations in a single environmental medium are insufficient to characterize risk transmission because MeHg formation, bioavailability, trophic transfer, and accumulation depend on interacting environmental and ecological processes. Second, food-web structure and transfer pathways are critical for identifying the biological groups and links that contribute most strongly to MeHg movement toward edible products. Third, network analysis, BNs, and Monte Carlo simulation provide complementary functions for pathway identification, probabilistic updating, and exposure-uncertainty analysis, respectively. Based on these findings, we propose a conceptual Network–BN–Monte Carlo framework linking pathway identification, probabilistic inference, and dietary-exposure uncertainty within a common analytical approach. The framework currently serves primarily to organize existing evidence; practical risk assessment and early warning will require empirical parameterization and independent validation using real monitoring data.

## 1. Introduction

Lakes, reservoirs, and aquaculture waters are affected by multiple pollution sources, including industrial discharges, agricultural non-point source pollution, and aquaculture activities, while chemical contaminants entering aquatic environments can undergo transport and transformation among the water column, suspended particulate matter, and sediments, and can subsequently be taken up and accumulated by aquatic organisms [1]. However, contaminant concentrations in environmental media do not directly reflect contamination levels in fishery products. Contaminant accumulation in fishery products is also influenced by contaminant speciation, bioavailability, species, habitat conditions, and feeding relationships [2,3,4]. Once fishery products are consumed, the resulting level of dietary exposure is further influenced by factors such as consumption amount, consumption frequency, and body weight [5]. Therefore, assessing food safety risks in fishery products arising from aquatic environmental contamination requires an integrated consideration of the continuous transfer of contaminants from environmental media through aquatic organisms and food webs to human exposure.

Mercury (Hg) and its organic form, methylmercury (MeHg), provide a representative example of the cross-medium risk transfer process described above. Inorganic Hg entering aquatic environments can migrate and exchange among the water column, particulate matter, and sediments, and can be converted to MeHg through microbial methylation under favorable environmental conditions [2,6,7]. Therefore, the risk associated with Hg/MeHg depends not only on Hg concentrations in aquatic environments but also on multiple factors, including methylation conditions, food-web structure, fishery product species, and human consumption patterns [3,5]. In addition, Hg speciation and its binding to dissolved organic matter (DOM) can affect Hg bioavailability [2,4], thereby influencing subsequent methylation processes. Methylating microorganisms and the associated *hgcAB* genes can indicate potential Hg methylation capacity, whereas actual MeHg production is jointly influenced by microbial activity, gene expression, and the availability of bioavailable Hg [8]. The resulting MeHg can subsequently be transferred to higher trophic levels through pelagic and benthic food webs, where it undergoes bioaccumulation and trophic magnification in edible fishery products such as fish [3,9,10]. Consumption of fishery products is an important route of dietary MeHg exposure in humans, and MeHg is a developmental neurotoxicant whose long-term excessive intake may adversely affect human health [5,11,12]. Therefore, this study focuses on MeHg in the assessment of food safety risks, and in dietary exposure assessments of MeHg, total mercury (THg) should not be directly used as a substitute for MeHg when speciation measurements or a reliable conversion basis is unavailable [1,5,11]. Data on Hg/MeHg in existing studies are derived from direct measurements, data conversion, and model predictions. When establishing a global database of Hg in fishery products, Zhou et al. included both measured and predicted MeHg concentrations [13]. The climate change scenario study by Wu et al. relied on model predictions [14], whereas Boldrocchi et al. directly measured THg and MeHg concentrations in the muscle tissues of multiple shark species [15]. Therefore, in the subsequent synthesis and comparison of relevant evidence, this review distinguishes both Hg species and data sources, explicitly differentiating among total THg, inorganic Hg, and MeHg, as well as among measured, converted, and model-predicted data.

Existing studies have accumulated substantial evidence on the environmental sources of Hg, its transport across environmental media, microbial methylation, bioavailability, food-web bioaccumulation, and dietary exposure [1,2,5,7,10,16]. However, differences in sampling periods, study regions, and assessment metrics make it difficult to directly compare and align the reported findings across studies. Contaminant concentrations in environmental media or biological tissues primarily reflect the contamination status at a particular stage and do not directly indicate the actual contribution of different transfer pathways to contamination in higher-trophic-level fishery products. By quantifying Hg fluxes through the Colorado River food web, Walters et al. found that Hg inputs to fish were concentrated in a limited number of feeding links, whereas some biological groups with high Hg inputs could act as “dead ends” in Hg transfer because of the absence of effective predator–prey linkages [17]. These findings indicate that identifying critical stages in Hg/MeHg risk transfer requires consideration not only of contaminant concentrations but also of food-web structure and the actual transfer processes involved.

Network analysis, Bayesian networks (BNs), and Monte Carlo simulation can support multi-stage risk analysis from different perspectives. Network analysis is primarily used to characterize transfer relationships among nodes and to identify key biological groups and risk propagation pathways. Existing studies have combined food-web feeding relationships with Hg fluxes to identify major Hg transfer pathways [17], while others have used graphical models to examine associations among different Hg species and related variables [18]. However, these approaches primarily characterize statistical relationships among variables and do not directly indicate the direction of Hg transfer or the actual magnitude of Hg fluxes. In contrast, BNs have been used to integrate information on environmental conditions, food webs, and contaminant monitoring, and to update the probabilities of different risk states as new environmental or monitoring evidence becomes available [19,20]. Monte Carlo simulation is primarily used to account for uncertainty in parameters such as fish MeHg concentrations, consumption rates, and body weight, thereby generating probability distributions of dietary exposure [21,22]. Existing studies suggest that these methods have mainly been applied to different stages of the risk chain, while their integrated application still requires further validation.

Against this background, this study adopts a structured narrative review approach, using Hg/MeHg as representative contaminants to examine how aquatic environmental contamination ultimately affects food safety risks associated with fishery products. The review first summarizes the transport and methylation of Hg across different environmental media, the regulatory effects of environmental factors such as eutrophication and algal blooms, and the mechanisms by which Hg accumulates through pelagic and benthic food webs in edible fishery products such as fish and ultimately contributes to human dietary exposure. The review then discusses the application of network analysis to characterize risk relationships and identify key nodes and transfer pathways, as well as the applications and limitations of BNs in multisource information integration, uncertainty representation, scenario analysis, and probabilistic early warning. Compared with reviews that focus separately on environmental cycling and methylation, food-web transfer, or dietary exposure, this review organizes the available evidence along a continuous risk chain and further examines the integrated application of network-based pathway identification, Bayesian network probabilistic inference, and Monte Carlo analysis of exposure uncertainty. This review also proposes an integrated network–probabilistic framework to organize existing mechanistic and pathway evidence, identify critical stages of risk transmission, and support risk management. At present, this framework primarily represents a conceptual integration based on existing evidence, and its further application will require parameterization with empirical data and independent validation.

## 2. Methods

### 2.1. Review Design and Literature Search

This study adopted a structured narrative review approach to organize evidence along the process of Hg/MeHg transport and transformation in aquatic environments, food-web transfer, contamination of fishery products, and subsequent human dietary exposure. Literature searches were conducted primarily in the Web of Science Core Collection, Scopus, PubMed, Europe PMC, and China National Knowledge Infrastructure (CNKI). The reference lists of relevant studies were also checked to identify potentially missed publications. The search focused primarily on studies published between 2000 and 2026, while seminal studies were retained where necessary. Official regulatory and guidance documents were additionally identified from the websites of the relevant national and international organisations and were retained in the final synthesis. Both English- and Chinese-language publications were included in the literature search.

The search terms mainly covered Hg/MeHg speciation and bioavailability, Hg methylation and *hgcAB*, food-web transfer and bioaccumulation, contamination of fishery products and dietary exposure, and Hg speciation analysis. For methodological evidence related to network analysis, Bayesian networks, and Monte Carlo simulation, targeted searches were conducted using method-specific keywords, and the reference lists of relevant studies were checked where necessary.

### 2.2. Literature Screening and Selection

**Figure 1:**
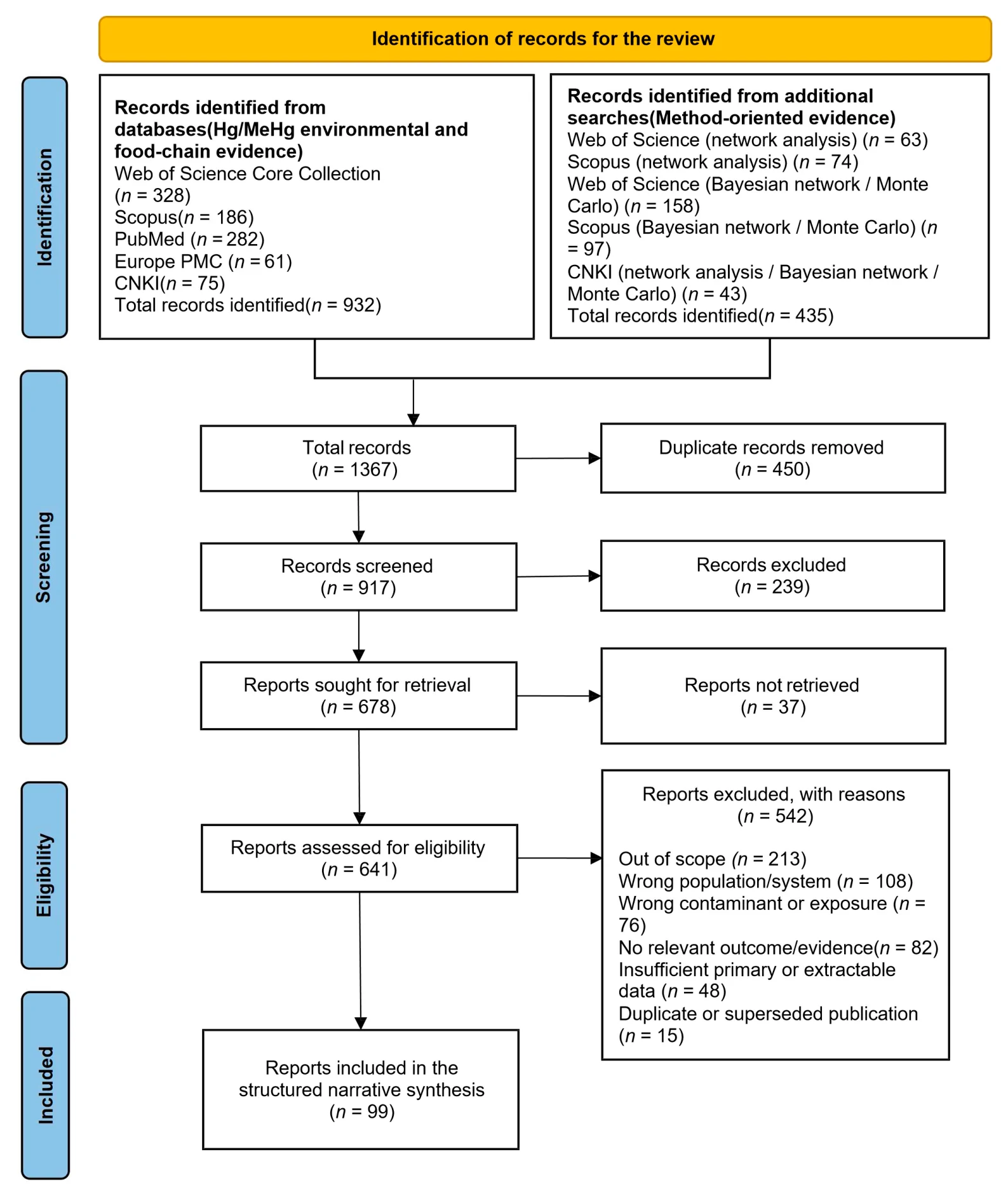
PRISMA-based literature identification and screening process for this structured narrative review. Adapted from the PRISMA 2020 framework. Official regulatory and guidance documents (*n* = 10) were identified from organisational websites and are included in the final count (*n* = 99).

The retrieved records were first screened based on titles and abstracts, and studies within the scope of this review were subsequently subjected to full-text eligibility assessment. Particular attention was given to studies directly addressing Hg/MeHg in aquatic environments, food webs, fishery products, or dietary exposure, as well as studies providing mechanistic, experimental, monitoring, or modeling evidence relevant to network analysis and probabilistic risk assessment. Studies were excluded if they were unrelated to the review topic, did not address the research questions of this review, lacked usable study information, or represented duplicate publications. For the included studies, information on the study system, Hg species, sample type, analytical methods, and major findings was extracted, and the results were compared in consideration of the specific conditions of field investigations, experimental studies, and modeling studies. This review focuses primarily on freshwater systems, while also considering estuarine and marine studies that provide evidence relevant to key mechanisms, methodological approaches, and exposure assessments of fishery products. The literature identification and screening process is shown in Figure 1.

## 3. Aquatic Environmental Contamination and Food Safety Risks in Fishery Products

The relationship between aquatic environmental contamination and chemical hazards in fishery products depends on contaminant transport, biological uptake, food-web accumulation, and dietary exposure. Together, these interconnected processes provide the basis for investigating Hg/MeHg risk transmission.

### 3.1. Aquatic Environmental Contamination and Chemical Hazards in Fishery Products

Pollutants in lakes, reservoirs, and aquaculture waters originate primarily from industrial discharges, municipal wastewater, agricultural non-point source inputs, and aquaculture activities. After entering aquatic environments, nutrients, heavy metals, and organic contaminants can be distributed among the water column, suspended particulate matter, and sediments, and their transport and transformation across these media can alter contaminant distribution and bioavailability, ultimately affecting the pathways and magnitude of exposure in aquatic organisms [23].

The water column, particulate matter, and sediments play distinct roles in this process. Dissolved contaminants can be transported with the water column, whereas particle-bound contaminants can enter sediments through adsorption and settling, making sediments important reservoirs for nutrients, heavy metals, and persistent organic pollutants. However, contaminants do not remain permanently sequestered in sediments and can be released back into the overlying water through processes such as wind- and wave-induced disturbance, sediment resuspension, bioturbation, and material exchange across the sediment–water interface. Because shallow lakes are characterized by relatively low water depths and frequent wind- and wave-induced disturbance, exchanges among these environmental media are particularly important for contaminant redistribution [23].

Water quality assessment, regulatory compliance assessment of contaminants in food, and human dietary exposure assessment differ in both their evaluation indicators and objectives. China’s Environmental Quality Standards for Surface Water (GB 3838—2002) [24] are primarily intended for the assessment of surface water quality and cannot be directly applied to determine the food safety of edible tissues from fishery products. Compliance assessment of contaminants in fishery products should be based on the corresponding food regulatory limits, whereas assessment of human health risks further requires dietary exposure evaluation that takes into account factors such as consumption amount, consumption frequency, and body weight. Internationally, the Codex Alimentarius Commission has established maximum levels for MeHg in certain fish species under the General Standard for Contaminants and Toxins in Food and Feed (CXS 193-1995) [25]. The European Commission and the United States Food and Drug Administration have also established corresponding maximum levels or action levels for Hg/MeHg in fishery products [26,27]. China’s current National Food Safety Standard—Maximum Levels of Contaminants in Foods (GB 2762—2025) specifies maximum levels for MeHg in aquatic animals and their products [28]. Regulatory frameworks differ in terms of the Hg species addressed, the fishery products covered, and the regulatory approaches adopted; therefore, compliance assessments of contaminants in food should be interpreted according to the specific scope of each framework. To compare food regulatory requirements for Hg/MeHg in fishery products and the reference values used for dietary exposure assessment across different regulatory systems, Table 1 summarizes representative food regulatory limits and health-based guidance values.

These comparisons show that regulatory limits for Hg/MeHg are not fully consistent across different regulatory frameworks. Compliance of contaminant concentrations in fishery products with regulatory requirements is assessed against food regulatory limits, whereas health-based guidance values are used to evaluate human dietary exposure over a defined period. Therefore, when comparing Hg/MeHg risks across regions or studies, the Hg species measured, the applicable food categories, and the nature of the assessment criteria should all be considered, and food regulatory limits should not be conflated with human health-based guidance values.

After entering aquatic environments, Hg can migrate among the water column, particulate matter, and sediments and can be methylated by microorganisms to form MeHg. MeHg is highly bioavailable and can undergo bioaccumulation and trophic magnification in aquatic food webs, ultimately leading to dietary exposure through the consumption of fishery products. Hg/MeHg therefore provides a representative example of the continuous process linking environmental transport and transformation, food-web transfer, contamination of fishery products, and human exposure. Accordingly, this review uses Hg/MeHg as representative contaminants to examine how aquatic environmental contamination can ultimately affect the food safety of fishery products. Chen et al. investigated small fish, crabs, mussels, and worms from 10 estuaries in the northeastern United States and found that MeHg concentrations in fish were associated with MeHg in suspended particulate matter, whereas MeHg concentrations in worms were associated with MeHg in sediments. By incorporating information on food sources and trophic position, the authors further examined these differences, indicating that MeHg accumulation in different biological groups may be linked to distinct environmental sources and transfer pathways [3].

In studies of Hg/MeHg in fishery products, directly measured concentrations should be distinguished from estimates derived from proportional conversion. Direct speciation analyses have shown that the proportion of MeHg varies among species and tissues. Zhang et al. found that both Hg concentrations and the proportion of MeHg varied among tissue types and categories of fishery products collected from coastal cities in China [30]. Hernández-Mendiola et al. also performed Hg speciation analysis in the muscle tissues of three fish species from markets in Mexico City and observed interspecific differences in the proportion of MeHg [31]. In contrast, Dogruyol et al. measured THg concentrations in farmed mussels from Türkiye and Bulgaria and estimated MeHg intake using proportions reported in the literature [32]. A meta-analysis by Eccles et al. further showed that MeHg/THg ratios vary among different foods and tissues, indicating that when MeHg is estimated from THg, conversion ratios should be selected to match the target food matrix [16].

### 3.2. Hg/MeHg Determination and Speciation Analysis

The analytical methods used for THg and MeHg in fishery products differ. THg is typically determined after sample homogenization and acid digestion using techniques such as inductively coupled plasma mass spectrometry (ICP-MS) or atomic absorption spectrometry, whereas MeHg requires extraction under conditions that preserve its chemical form as far as possible, followed by chromatographic separation and detection for speciation analysis [31,33,34]. When comparing results across studies, attention should be paid to the sample matrix, edible portion, processing status, wet- or dry-weight basis, analytical methods, and quality-control conditions, and information such as recovery, limit of detection (LOD), and limit of quantification (LOQ) should also be checked when necessary. When dry-weight concentrations are converted to a wet-weight basis, the moisture content used for the conversion should be specified. THg can be used for regulatory screening in certain food assessments; for example, under relevant Codex standards, THg may serve as a screening parameter to determine whether further MeHg analysis is warranted [25]. For dietary exposure assessment of MeHg, directly measured MeHg concentrations should be used preferentially, and when MeHg is estimated from THg, the basis for the conversion should be clearly stated.

### 3.3. Risk Transmission from Aquatic Environmental Contamination to Dietary Exposure Through Fishery Products

For clarity in the following discussion, bioavailability refers to the extent to which Hg/MeHg can be taken up by organisms; bioaccumulation refers to the accumulation of contaminants within organisms through pathways such as water and diet; and biomagnification refers to the increase in contaminant concentrations along predator–prey relationships or with increasing trophic level [2,10,35].

Aquatic organisms can take up contaminants directly from the surrounding environment or through dietary intake, but contaminant concentrations in the environment do not directly represent the fraction that is actually available for biological uptake. The chemical speciation of contaminants and their binding to environmental media can influence their bioavailability. Seelen et al. conducted short-term uptake experiments with marine diatoms under different dissolved organic matter conditions and found that the binding of MeHg to organic matter affected MeHg uptake by algal cells [4]. Shen et al. further showed, through competitive binding experiments between MeHg–cysteine and natural DOM, that the abundance of thiol groups in DOM affects ligand-exchange kinetics and, over time, alters the adsorption and uptake of MeHg by cyanobacteria [36]. These findings indicate that dissolved organic carbon (DOC) concentration alone is insufficient to fully characterize the bioavailability of MeHg. Therefore, when assessing contaminant accumulation in fishery products on the basis of environmental contamination levels, contaminant speciation, bioavailability, species-specific habitat, and feeding behavior should also be taken into account.

Once contaminants enter the food web, they can accumulate in organisms and be transferred to higher trophic levels through predator–prey interactions, ultimately reaching fishery products consumed by humans. After contaminants are ingested by humans, the risk chain extends further to dietary exposure. Factors such as consumption amount, consumption frequency, body weight, and age can all influence actual intake levels; therefore, contaminant concentrations below the corresponding regulatory limits do not necessarily indicate a low risk from long-term dietary exposure. Dietary exposure assessment also requires the selection of appropriate toxicological reference values according to the chemical form of the contaminant, together with consideration of population-specific consumption parameters [11,12].

For Hg/MeHg, contamination levels in environmental media, accumulated concentrations in biological tissues, and human dietary intake correspond to different stages of the risk transmission chain. The following sections further discuss the environmental transport and transformation of Hg/MeHg, its transfer through food webs, and subsequent dietary exposure.

## 4. Transport and Transformation of Hg/MeHg Across Lake Environments, Food Webs, and Human Exposure

Environmental conditions and ecological processes regulate Hg/MeHg transformation, accumulation within food webs, and dietary exposure. This section examines the interconnections among environmental sources, methylation, environmental regulation, food-web transfer, and exposure under different environmental conditions.

### 4.1. Environmental Sources, Methylation, and Exchange of Hg/MeHg Among Environmental Media

Hg can enter lakes through atmospheric deposition, watershed runoff, and point-source discharges, and can undergo adsorption, settling, release, transport, and transformation among the water column, particulate matter, and sediments [1]. Sediments can store a portion of Hg and can also influence Hg distribution in the overlying water through exchange across the sediment–water interface [23]. Sediments are also important sites for the accumulation of particulate Hg and the conversion of inorganic Hg to MeHg, with certain microorganisms capable of methylating divalent inorganic Hg to form MeHg [6]. Therefore, THg concentrations alone cannot fully reflect differences in Hg methylation and MeHg formation.

Parks et al. demonstrated, through deletion and complementation experiments targeting the *hgcA* and *hgcB* genes in two model microorganisms, that both genes are essential for Hg methylation [6]. Therefore, the detection of *hgcAB* genes in environmental samples can be used to assess potential Hg methylation capacity, but actual methylation rates cannot be inferred solely from their presence or abundance [8]. Capo et al. simultaneously measured *hgc* genes and their transcripts, Hg species, and isotope-tracer-derived methylation rate constants in stratified water columns at two sites in the Baltic Sea. The results showed that although *hgc* genes and transcripts were detected in some redox transition zones, methylation rates were low or undetectable, whereas incorporating information on transcript abundance and dissolved divalent inorganic Hg sulfide species provided a better explanation for variations in methylation rates and MeHg concentrations [8]. Peterson et al. analyzed *hgc*-related genes and transcripts in eutrophic Lake Mendota and measured Hg methylation rates in the water column using tracer incubation experiments. The study found that Hg methylation rates decreased markedly after molybdate was added to inhibit sulfate reduction, suggesting that sulfate-reducing microorganisms may contribute to Hg methylation in the anoxic water layer of the lake [37]. Gionfriddo et al. found in culture experiments that higher *hgcA* expression did not necessarily result in greater MeHg production [38]. This finding indicates that gene expression can reflect microbial methylation potential, whereas actual MeHg production is also influenced by factors such as Hg bioavailability. Therefore, evaluation of actual methylation levels should integrate *hgcAB* gene expression with directly measured methylation rates.

Recent studies have reported similar findings. A metagenomic analysis across freshwater systems found that *hgcA* was more abundant under low-oxygen conditions, indicating that microorganisms with Hg-methylating potential are more prevalent in such environments [39]. However, these results primarily reflect the distribution of Hg-methylating microorganisms and do not directly indicate actual MeHg production. Another study of aquaculture pond sediments combined isotope tracing, inhibition experiments, and functional gene analysis and found a positive correlation between *hgcAB* abundance and Hg methylation rates [40]. This finding indicates that functional genes can correspond well with actual methylation processes under specific environmental conditions, although the strength of this relationship may vary among aquatic systems and environmental settings.

In addition to the intrinsic methylation potential of microorganisms, external inputs may also alter the conditions governing MeHg production. By combining a metagenomic survey of urban rivers with wastewater-amendment experiments, Xia et al. found that wastewater inputs enhanced MeHg production under deoxygenated incubation conditions with added Hg tracers, and that the presence of sediments influenced this process [41]. However, such incubation experiments primarily provide evidence of potential mechanisms and cannot be used directly to infer actual contamination levels in natural rivers or fishery products.

In addition, dissolved oxygen (DO), redox conditions, organic matter, and Hg speciation can all influence the activity of Hg-methylating microorganisms and the bioavailability of Hg [2]. The study by Capo et al. showed that the redox state of the water column was not linearly related to net MeHg accumulation, and that Hg methylation rates and MeHg accumulation were jointly influenced by Hg speciation and other environmental conditions [8]. Fuhrmann et al. also observed differences in net MeHg accumulation under different redox conditions in sediment–water interface microcosm experiments [42]. Similarly, four years of geochemical profiling and metagenomic data from Brownlee Reservoir showed that MeHg production may also occur in the water column under nitrate- or manganese-reducing conditions [43]. In addition to redox conditions, the composition of organic matter and its binding characteristics with Hg can also influence Hg methylation. DOM comprises a variety of organic components, whereas DOC primarily reflects the carbon content of dissolved organic matter. The study by Seelen et al. showed that DOC concentration alone cannot fully capture the effects of organic matter on Hg bioavailability [4]. Other sediment studies have also found that microbial community composition and the partitioning of Hg between the solid phase and porewater can influence Hg methylation processes [44,45]. In culture experiments with *Geobacter sulfurreducens*, Gutensohn et al. observed that cysteine initially enhanced Hg uptake and methylation, but this promoting effect gradually weakened as cysteine was transformed into penicillamine [46]. These findings indicate that the composition and transformation of organic ligands can also influence Hg bioavailability and MeHg production.

Sediments are important sites of Hg methylation, but MeHg can also be produced within the water column [2,7,47]. By combining tracer incubations of water and sediments with model analysis in Nacimiento Reservoir, Millard et al. found that during summer stratification and anoxia, water-column methylation may represent an important source of MeHg in bottom waters [47]. Capo et al. also measured Hg methylation rates in a stratified brackish water column, providing further evidence that MeHg can be produced within the water column [8]. These studies indicate that the relative contributions of sediments and the water column to MeHg production may vary with water-column structure and seasonal conditions. Gallorini and Loizeau found that THg and MeHg concentrations in lake-snow particles from Lake Geneva increased with depth and, based on mass-balance analysis, proposed that particle interiors may provide microenvironments favorable for Hg methylation [48]. This finding provides indirect evidence for Hg methylation within particles.

### 4.2. Effects of Eutrophication, Algal Bloom Succession, and CO_2_ Changes on Hg/MeHg

Eutrophication can alter algal biomass, organic matter supply, and oxygen conditions in the water column, thereby affecting Hg methylation and the pathways through which MeHg enters food webs. Existing studies have investigated the relationships between eutrophication and Hg/MeHg from different perspectives, including field monitoring and laboratory incubation experiments [49,50].

During periods of rapid algal growth, changes in algal density can affect the partitioning of Hg/MeHg between the water column and plankton. Li et al. investigated 17 sampling sites in Lake Taihu and found that areas with high algal density had relatively lower THg bioconcentration factor (BCF) in phytoplankton, THg bioaccumulation factor (BAF) in zooplankton, and proportions of MeHg in THg within plankton [51]. The authors attributed this pattern mainly to differences in zooplankton body size, higher pH in areas with greater algal density, and lower Hg bioavailability [51]. After analyzing MeHg, dissolved organic carbon (DOC), and plankton of different size fractions in the Great Lakes, Ogorek et al. found that phytoplankton exhibited higher MeHg bioaccumulation factors in waters with lower DOC concentrations [52]. These findings indicate that the effects of algal growth on Hg/MeHg accumulation depend not only on algal density but also on interacting factors such as pH, DOC, and biological community characteristics.

In addition, algal-derived organic matter may also influence MeHg production. Lei et al. conducted anaerobic dark-incubation microcosm experiments using sediments from 10 lakes and found that, at an algal addition level of 1000 mg/L after 21 days of incubation, sediment MeHg concentrations were 54–514% higher than those in the corresponding controls without algal addition [49]. However, this result reflects an experimental effect under specific incubation conditions and should not be directly interpreted as the magnitude of change occurring before and after algal blooms in natural lakes. In contrast, field observations indicate that changes in algal biomass do not correspond in a simple manner to MeHg levels. Balzer et al. found in Lake Ölper, Germany, that the proportion of MeHg in THg within suspended particles larger than 25 μm reached a maximum of approximately 26% at the oxic–suboxic boundary, while the period of peak algal biomass did not coincide with the months of elevated MeHg concentrations [50]. Lei et al. subsequently found in Hg(II)-spiked lake-water microcosm experiments that, under anaerobic dark-incubation conditions, the addition of algal-derived organic matter resulted in higher dissolved MeHg concentrations than those in controls using the same lake water and Hg addition but without algal amendment [53]. These findings indicate that algal-derived organic matter may contribute to MeHg production, although its effects depend on the environmental medium and specific environmental conditions.

Following algal senescence and decomposition, the composition and properties of algal-derived organic matter also change. Li et al. conducted microbial degradation experiments using different sources of algal-derived DOM and further examined their effects on MeHg uptake by *Microcystis*. The results showed that decreases in DOC were not always accompanied by corresponding changes in MeHg uptake [54], indicating that the effects of algal-derived organic matter on MeHg bioavailability depend not only on its quantity but also on changes in organic matter composition. Isotope-tracer experiments in lake water further showed that the effects of DOM vary according to its source. Compared with terrestrial DOM, algal-derived DOM exerted a stronger stimulatory effect on Hg methylation but a weaker effect on demethylation, thereby favoring net MeHg production [55]. This indicates that the effects of organic matter on net MeHg accumulation also depend on its source and on the balance between methylation and demethylation processes.

In addition to algal succession, changes in CO_2_ may also affect MeHg production and accumulation, although findings across studies have been inconsistent. Wu et al. found in a rice–fish–snail wetland microcosm experiment that elevated CO_2_ increased MeHg concentrations in the water and in some organisms [56]. In contrast, Lei et al. conducted microcosm experiments using water samples from 45 freshwater lakes and found that, compared with 420 ppm CO_2_, net MeHg production under 1000 ppm CO_2_ decreased by 14–96%, with an average reduction of 63% [57]. The two studies differed in biological composition, Hg input conditions, and assessment metrics, indicating that the effects of CO_2_ on MeHg processes may vary depending on the specific environmental and experimental conditions.

In natural waters, MeHg concentrations can also vary seasonally and with water-column stratification. Armstrong et al. conducted seasonal sampling of water and suspended particles in Lakes Mendota and Monona and found that MeHg concentrations varied across seasons and water-column depths. Based on concentration measurements and stable-isotope data, the authors concluded that water-column-derived MeHg made an important contribution to the local food web [58]. Fuhrmann et al. found in Hodges Reservoir that MeHg inputs to the water column exhibited pronounced seasonal variation, which may be related to sediment release and in situ MeHg production within the water column [59]. Zhu et al. likewise found in the Dongtan wetland of the Yangtze River Estuary that the seasonal variations in THg and MeHg were not fully consistent [60]. A study of six seasonally ice-covered lakes in northern China further found that MeHg/THg ratios were higher during the ice-covered period, whereas the absolute concentrations of both THg and MeHg were generally higher during the ice-free period [61]. These findings indicate that MeHg/THg ratios and absolute concentrations can exhibit different seasonal patterns, with the MeHg/THg ratio primarily reflecting relative changes in Hg speciation. Overall, MeHg dynamics under eutrophic and algal-bloom conditions are jointly influenced by organic matter characteristics, water-column stratification, seasonal variation, and site-specific ecological conditions.

### 4.3. Bioaccumulation and Trophic Magnification in Aquatic Food Webs

MeHg can enter aquatic food webs through both pelagic and benthic pathways. In the pelagic pathway, dissolved MeHg or MeHg associated with suspended particles is taken up by phytoplankton and zooplankton and is subsequently transferred to small fish and predatory fish. In the benthic pathway, MeHg in sediments or near the sediment–water interface enters benthic invertebrates and is subsequently transferred to benthic, omnivorous, or predatory fish [3,4]. However, MeHg transfer pathways in natural food webs are generally more complex. Jonsson et al. added Hg isotope tracers to the water column and to different sediment locations in an estuarine mesocosm experiment and found that MeHg accumulation in benthic fauna varied with the location and chemical form of Hg, as well as among faunal groups [62]. Hall et al. combined diel sampling with biomass and stable-isotope data in Lake Katepwa, Canada, and proposed that the vertical migration of adult *Leptodora kindtii* may transfer MeHg from deeper waters into the feeding pathways of planktivorous fish [63]. In addition, field surveys and bioenergetic modeling by He et al. showed that grass shrimp can serve as a link between sediment-associated food sources and juvenile fish, with their estimated contribution varying according to the dietary composition of juvenile fish [64]. Therefore, identifying the major MeHg transfer pathways within food webs requires integrated consideration of contaminant location, organismal movement, and specific feeding relationships.

When describing Hg accumulation between environmental media and organisms, the BCF, BAF, and biota–sediment accumulation factor (BSAF) should be distinguished. BCF primarily reflects the extent of direct contaminant uptake from water, whereas BAF also incorporates other exposure pathways, including dietary uptake [35]. BSAF is used to compare contaminant concentrations in sediments and biota; its calculation requires clear specification of the Hg species, tissue type, wet- or dry-weight basis, and normalization approach, while ensuring that the same Hg species is compared in both matrices [62]. Under natural environmental conditions, Hg accumulation in organisms is generally influenced by multiple exposure pathways, including direct uptake from water and dietary uptake. In feeding-tracer experiments with oysters, Metian et al. found that the assimilation efficiencies of MeHg and inorganic Hg differed when oysters were fed ciliates or diatoms, and that the results also varied among food types [65]. These findings indicate that both Hg species and food type can influence Hg transfer to consumers.

The biomagnification factor (BMF) primarily describes the concentration relationship between a specific predator and its prey, whereas the trophic magnification factor (TMF) reflects the overall trend in contaminant concentrations with increasing trophic level across an entire food web [10]. Wang et al. found in a food-web study of Lake Taihu that both THg and MeHg concentrations increased with trophic level [9]. However, even among organisms occupying similar trophic levels, MeHg concentrations can still be influenced by food sources and habitat conditions [3,10]. A meta-analysis of freshwater predatory invertebrates by Sinclair et al. further showed that the magnitude of Hg biomagnification varies among taxonomic groups, feeding guilds, and methods used to estimate trophic level [10]. A field study in the Bohai Sea further found that MeHg biomagnified with increasing trophic level, whereas inorganic Hg exhibited trophic dilution, and that MeHg concentrations in fish were also influenced by factors such as species, body weight, and feeding habits [66]. Therefore, analyses of Hg transfer through food webs should consider not only trophic level but also specific species, feeding relationships, and habitat conditions.

Such differences are also evident among different aquatic systems and food webs. Gentès et al. observed trophic magnification of MeHg in all four freshwater lakes studied in France, although the trophic magnification slopes differed among lakes [67]. Chiang et al. compared food webs from Patagonia and the Antarctic coast and found that sites closer to freshwater inputs exhibited steeper trophic magnification slopes, while the calculated slopes also varied depending on the biological groups included in the analysis [68]. These studies indicate that the extent of MeHg trophic magnification is influenced by study region, food-web composition, and the organisms sampled, and therefore trophic magnification slopes may differ among aquatic systems.

In addition to food-web structure, individual fish size can also influence MeHg concentrations in edible tissues. Polak-Juszczak measured THg and MeHg in the muscle tissue of *Belone belone* from the southern Baltic Sea and found that both were positively correlated with body length, body weight, and age [69]. Hu et al. also observed positive correlations between MeHg concentrations and both body length and body weight in commercially important fish species from the Erhai Lake basin [70]. These findings indicate that body length, body weight, and age are important individual-level variables when comparing monitoring data and exposure within the same fish species. Comparisons across species and aquatic systems should also take into account differences in growth characteristics, feeding habits, and life-history traits. Habitat conditions may further contribute to these differences. Romero-Romero et al. performed Hg speciation analysis in the muscle tissues of 25 fish species from the Cantabrian Sea and found that THg and MeHg concentrations generally increased with habitat depth, while demersal fish also exhibited higher THg concentrations than pelagic fish within the same depth range [71]. Therefore, MeHg concentrations in fish are related not only to individual size but also to species-specific traits and habitat conditions.

Benthic invertebrates play an important role in linking sediments and water-column food sources to fish. Both the estuarine survey by Chen et al. and the tracer experiment by Jonsson et al. showed that differences in food sources among biological groups can influence their MeHg accumulation [3,62]. Such studies are primarily used to elucidate MeHg transfer pathways within food webs, whereas human dietary exposure assessments focus more directly on MeHg concentrations in the portions actually consumed. Chen et al. analyzed whole-body tissues of small fish, whereas Jonsson et al. used pooled dry-weight samples of benthic fauna [3,62]. These data are suitable for examining MeHg transfer within ecosystems, but dietary exposure assessment requires concentration data corresponding specifically to edible tissues. Table 2 summarizes the study systems, major experimental or environmental conditions, and key findings of the relevant studies.

### 4.4. Dietary Exposure Risk Assessment of MeHg from Fishery Products

Table 3 summarizes directly measured MeHg concentrations in edible fish and the main analytical methods reported in recent studies, including market fish from Italy, farmed fish from Norway, and market fish from Mexico City [31,33,72]. The table also lists both publication year and sampling year to reflect the temporal basis of the data used in each study.

As shown in Table 3, MeHg concentrations vary substantially among species and across different market or aquaculture systems. The studies also differ in sample size, pooling strategy, sampling year, and statistical metrics. Pooled samples can reflect the overall level for a given species or batch but provide limited information on individual variability. Therefore, when these data are used for dietary exposure assessment, the concentration parameters selected should be consistent with the corresponding sampling design and statistical form.

Studies on non-fish fishery products also indicate that species and sample portion can influence measured MeHg concentrations. Hansen et al. performed Hg speciation analysis on the soft tissues of three bivalve groups and found both interspecific and intraspecific variation in the proportion of MeHg in THg [73]. Multi-site monitoring of mussels and oysters along the Korean coast also revealed pronounced spatial differences, with higher Hg levels observed at some sites near industrial facilities [74]. Ra et al. separately measured THg and MeHg in muscle, head, and whole-body samples from 11 shrimp species and found that Hg concentrations differed among sample portions [75]. Therefore, concentration data used for dietary exposure assessment should, as far as possible, correspond to the portions actually consumed and the relevant consumption practices.

MeHg can enter the edible tissues of fishery products through food-web transfer and subsequently enter the human body through consumption. Dietary exposure assessment typically combines MeHg concentrations in edible tissues with population-specific parameters such as consumption amount, consumption frequency, and body weight to estimate human MeHg intake [11]. Measured MeHg concentrations corresponding to the actual edible portion should be used preferentially, and the units and weight basis of the concentration data should be consistent. THg represents the total amount of all Hg species in a sample, whereas inorganic Hg and MeHg have different toxicological significance; therefore, the concentration data and toxicological reference values used should correspond to the Hg species being assessed [11,12]. When MeHg concentrations are estimated from THg, the basis for the conversion should be clearly specified. The estimated daily intake (EDI) of MeHg can be calculated using the following equation [76]:

$$\mathrm{EDI}=\frac{\sum_{i=1}^{n}C_{i}\times IR_{i}\times EF_{i}}{\mathrm{BW}\times 365}$$

where *C_i_* is the MeHg concentration in the i-th aquatic product (mg/kg); *IR_i_* is the mean consumption rate of that product on actual consumption days (kg/d); *EF_i_* is the annual number of consumption days (d/year); *BW* is body weight (kg); and *n* is the number of aquatic product types included in the assessment. EDI is expressed in mg/(kg bw d). If consumption has already been converted to a long-term annual average daily intake, consumption frequency should not be applied again, to avoid double adjustment [76].

In addition, processing can affect contaminant concentrations in fishery products; therefore, the concentration data used in exposure assessment should, as far as possible, correspond to the actual form in which the products are consumed [77]. After the EDI is obtained, it can be compared with a reference dose corresponding to the relevant Hg species, units, and exposure time scale, and the target hazard quotient (THQ) can then be calculated [12,76]:

$$\mathrm{THQ}=\frac{\mathrm{EDI}}{\mathrm{RfD}}$$

The chronic oral reference dose (RfD) for MeHg adopted by the U.S. Environmental Protection Agency (EPA) is 1.0 × 10^−4^ mg/(kg bw·d), equivalent to 0.1 μg/(kg bw·d), and was derived primarily on the basis of adverse effects of prenatal MeHg exposure on fetal neurodevelopment [12]. This value is a health-based reference dose for human intake rather than a concentration limit for MeHg in food. A THQ ≤ 1 indicates that, under the specified exposure parameters and assessment model, the estimated intake does not exceed the corresponding reference dose. A THQ > 1 indicates that the estimated intake exceeds the reference dose, suggesting that potential health risks warrant attention, but it does not imply that adverse health effects will necessarily occur [12].

Food contaminant limits and health-based guidance values for human intake serve different purposes. The former are primarily used to determine whether contaminant concentrations in food comply with regulatory requirements, whereas the latter are used to assess human intake over a specified period. Dietary exposure assessment therefore also requires consideration of population-specific parameters such as food consumption, body weight, and age. For specific populations such as children, consumption parameters appropriate to their age and body weight should be used [11]. The provisional tolerable weekly intake (PTWI) for MeHg established by WHO/JECFA is 1.6 μg/(kg bw·week), and exposure assessment should compare this value with weekly intake normalized to body weight while also considering the greater susceptibility to MeHg during embryonic and fetal development [11]. The PTWI and the U.S. EPA RfD are expressed on weekly and daily time scales, respectively, and originate from different assessment frameworks; therefore, each should be applied according to its specific conditions of use [11,12]. Liu et al. combined species identification of shark meat from markets in Taiwan with directly measured MeHg concentrations and estimated weekly intake and hazard quotient (HQ) for different age groups of children, demonstrating that both food type and population-specific parameters can influence exposure assessment results [78].

Population characteristics such as consumption amount, consumption frequency, and body weight vary among individuals and groups; therefore, even when fishery products contain similar contaminant levels, actual exposure may differ across populations. In the German BfR MEAL Study, Sarvan et al. combined MeHg concentrations measured in pooled samples prepared according to local consumption practices with food consumption data and found that the estimated intake of some young high consumers exceeded the weekly health-based guidance value used in the assessment [79]. Chang et al. separately evaluated women of childbearing age and children in Taiwan, China, and distinguished between the total population and actual consumers, showing marked differences in upper-end exposure levels among population groups [80]. These studies indicate that, in addition to average consumption levels, high consumers and specific population groups also warrant separate consideration. At the same time, MeHg concentrations in fishery products also vary, and the use of a single mean value alone may not adequately represent the overall distribution of population exposure [22]. Therefore, when sufficient data are available, probabilistic approaches can be used to simultaneously account for variability in parameters such as contaminant concentration, consumption amount, consumption frequency, and body weight. Krupa et al. combined fish muscle monitoring data from the Eeyou Istchee region of Canada with residents’ consumption data to conduct a dietary exposure assessment [76]. On this basis, Monte Carlo simulation can be used to estimate the probability distributions and different percentiles of EDI or THQ, thereby characterizing potential exposure levels across different population groups [22,76]. A study of 501 women of childbearing age by Buchanan et al. further found only a weak correlation between Hg exposure estimated from dietary data and measured hair THg concentrations [81], indicating that dietary surveys, food concentration data, and biomonitoring can provide complementary perspectives on human Hg exposure.

**Figure 2:**
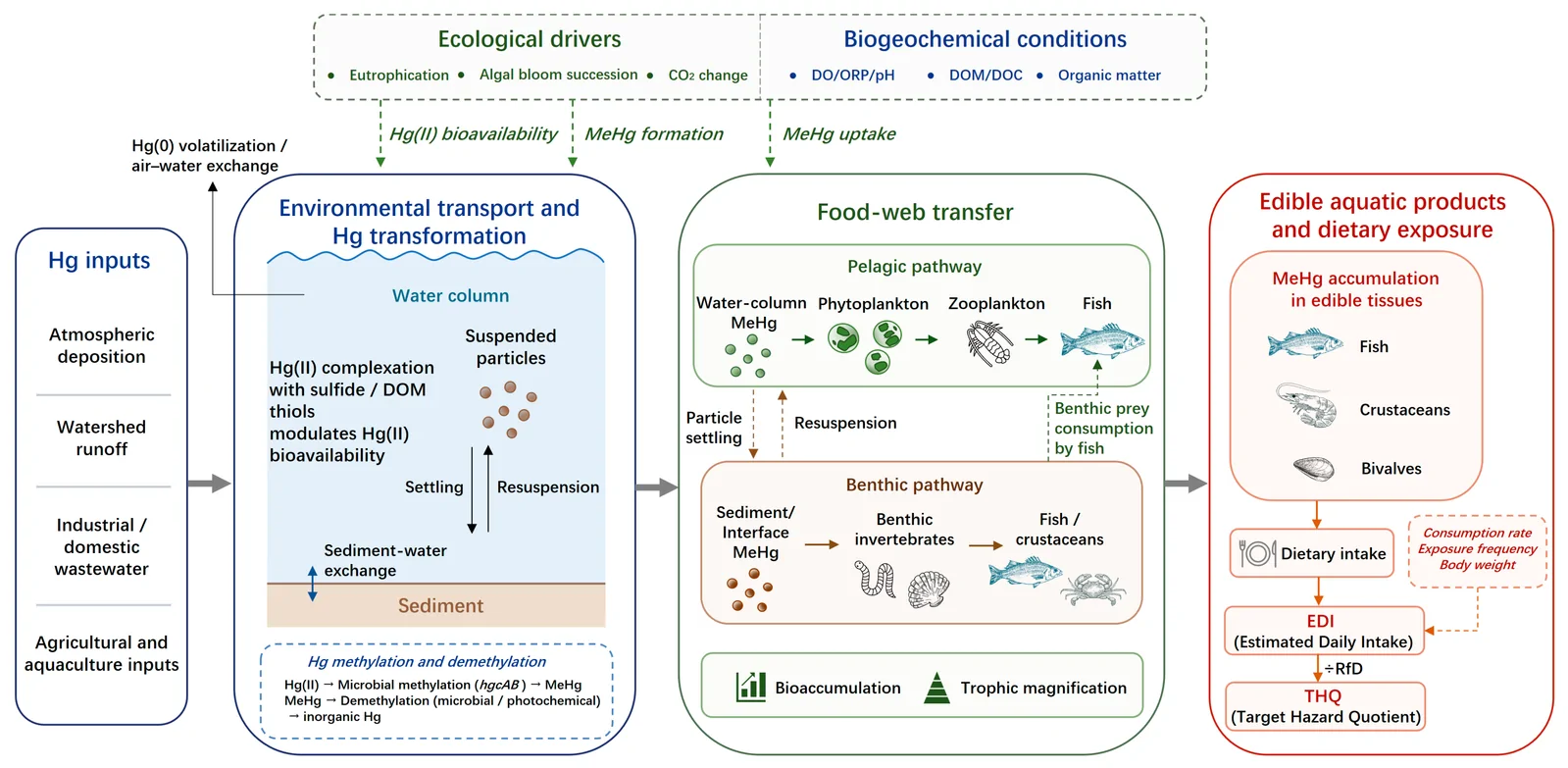
Framework for Hg/MeHg transfer from aquatic environments to dietary exposure. Exogenous Hg can enter aquatic environments through atmospheric deposition, watershed runoff, anthropogenic discharges, and other pathways, and can subsequently undergo transport and exchange among the water column, suspended particulate matter, and sediments. A fraction of inorganic Hg can be converted to MeHg through microbial processes, after which MeHg enters aquatic food webs through pelagic and benthic pathways and accumulates in fishery products. Consumption of fishery products subsequently results in dietary exposure, which can be assessed using indicators such as estimated daily intake (EDI) and target hazard quotient (THQ). Environmental conditions, including eutrophication, algal blooms, CO_2_, dissolved oxygen (DO), oxidation–reduction potential (ORP), pH, and DOM, can influence Hg/MeHg speciation and transformation, bioavailability, and food-web uptake, but do not directly represent the actual magnitude of Hg/MeHg transfer between different stages.

The results of probabilistic exposure assessment also depend on the input data and parameter settings. Lin et al. measured THg concentrations in raw and cooked fishery products from Taiwan, China, estimated MeHg concentrations using literature-derived conversion ratios, and subsequently applied Monte Carlo simulation to assess dietary MeHg exposure across different age groups [82]. When directly measured MeHg data are unavailable, this approach can provide exposure estimates, but the results are affected by the conversion ratio used to estimate MeHg from THg. Charette et al. further evaluated parameters including the proportion of MeHg, in vitro bioaccessibility, and cooking-induced weight loss, and found that different parameter settings altered the resulting exposure estimates [83]. A review by Gong et al. on the in vitro bioaccessibility of Hg in foods likewise showed that Hg species, food composition, cooking methods, and in vitro digestion conditions can all influence the measured results [84]. In this context, in vitro bioaccessibility primarily refers to the fraction of MeHg released from food into digestive fluids during simulated digestion and can be used to evaluate the extent to which foodborne MeHg becomes available during the digestive process. Overall, dietary exposure to MeHg is influenced not only by contamination levels in fishery products and population-specific consumption characteristics but also by the data sources and parameter settings used in the assessment. Integrating the environmental transport and transformation, food-web transfer, and dietary exposure processes discussed above, Figure 2 summarizes the major pathways and influencing factors through which Hg/MeHg is transferred from aquatic environments into fishery products and subsequently contributes to dietary exposure.

The concentrations, transformation rates, bioaccumulation metrics, and dietary intake doses associated with the individual stages shown in Figure 2 reflect different aspects of the Hg/MeHg transfer process. Therefore, no single indicator can fully characterize the entire process from aquatic environmental contamination to human exposure. On this basis, further identification of structural relationships among environmental media, biological groups, and exposure stages requires these processes to be represented within an analytical framework with clearly defined nodes and connections. The following sections further discuss the role of network analysis in identifying risk pathways and the applications of Bayesian networks and Monte Carlo simulation to uncertainty analysis and probabilistic risk assessment.

## 5. Application of Network-Based Methods to Risk Pathway Identification and Probabilistic Early Warning for Contamination in Fishery Products

The environmental transport and transformation of Hg/MeHg, food-web transfer, and dietary exposure processes described in Section 4 can be used to define the environmental media, biological groups, and exposure indicators that should be considered in network analysis. Network analysis is primarily used to characterize connections among nodes and identify potential risk propagation pathways, BNs enable probabilistic inference based on available evidence, and Monte Carlo simulation is used to examine how variability in input parameters affects the distribution of exposure outcomes [17,22,85]. These three approaches differ in the questions they address, the data they require, and the forms of their outputs; therefore, this section discusses their applications to Hg/MeHg risk analysis based on existing studies and further proposes a conceptual Network–BN–Monte Carlo framework.

### 5.1. Network Representation and Pathway Identification of Contamination Risk Transmission in Fishery Products

Network representation requires nodes, connections, and their meanings to be defined according to the specific research question. In the context of Hg/MeHg risk propagation, nodes may represent the water column, suspended particulate matter, sediments, and different biological groups, whereas connections may represent intermedium transport, feeding relationships, or other relationships supported by mechanistic and observational evidence [17,23]. Indicators such as dissolved oxygen (DO), oxidation–reduction potential (ORP), pH, total nitrogen (TN), total phosphorus (TP), and DOC primarily characterize environmental conditions and can be used to explain Hg transport and transformation processes, but their values do not directly represent the actual magnitude of Hg transfer between nodes. If these indicators are further incorporated into a Bayesian network (BN), the variable states, data sources, and relationships among variables must also be explicitly defined [85].

Different connections represent distinct processes and should therefore be quantified using process-specific metrics. Hg transport between environmental media can be represented by exchange fluxes or transfer rates, accumulation between environmental media and organisms can be characterized using indicators such as BCF, BAF, or BSAF, and Hg intake fluxes along predator–prey links can be estimated by combining dietary composition, consumption rates, and contaminant concentrations [10,17,62]. The BMF primarily reflects the concentration relationship between a specific predator and its prey, whereas the TMF is used to characterize the overall trend of trophic magnification across the entire food web. Therefore, when different processes are incorporated into a single network, the metrics used for each type of connection and their corresponding physical meanings should be clearly specified. Krause et al. combined water-column MeHg concentrations with streamflow to estimate riverine MeHg loads, demonstrating that further quantification of connections among environmental media requires corresponding hydrological and contaminant-monitoring data [86]. When temporal variation and lagged effects need to be considered, dynamic Bayesian networks can be further used to represent relationships among variables across different time steps [87].

Network structural metrics primarily reflect the connectivity characteristics of nodes within a predefined network. Nogales et al. constructed a food safety incident network using notifications from the Rapid Alert System for Food and Feed (RASFF) and analyzed the structural positions of different countries in the network using degree and betweenness centrality [88]. Tapia-Contreras et al. used a Bayesian undirected graphical model to examine statistical associations between tissue metal concentrations and body-size metrics [18]. These studies show that connections in different networks may represent distinct relationships, such as commodity flows, statistical associations, or contaminant transfer, and therefore similar network structures should not automatically be assigned the same meaning in terms of risk propagation. Nodes with high centrality may be structurally important, but their actual roles in Hg/MeHg transfer should still be evaluated together with information on contaminant concentrations, feeding relationships, and transfer fluxes.

The study by Walters et al. on the Colorado River food web in the United States provides an example of contaminant flux analysis. The researchers combined THg concentrations in food resources and consumers with dietary composition, production, and consumption rates to estimate annual THg fluxes across different trophic links [17]. The results showed that blackflies contributed 56–80% of Hg intake flux to fish through dietary pathways, whereas New Zealand mud snails, despite taking up substantial amounts of Hg, contributed little to Hg transfer to fish because they were rarely consumed by fish. Changes in food-web composition and feeding relationships induced by experimental floods further altered Hg fluxes to rainbow trout [17]. This study indicates that the Hg accumulation level of a node alone does not directly represent its contribution to downstream transfer, and that pathway identification also requires consideration of actual feeding relationships and contaminant fluxes. It should be noted that this study primarily quantified THg, and its results were also constrained by the sampling frequency of tissue Hg measurements and by assumptions associated with the relevant model parameters.

In addition to direct estimation of contaminant fluxes, information on food sources and stable-isotope analyses can also support the identification of major transfer pathways. Existing studies have shown that Hg accumulation in different biological groups is related to the environmental media and food sources they utilize [3,62]. Armstrong et al. and Yang et al. further used Hg stable isotopes or compound-specific isotope analysis of MeHg to compare the relative contributions of pelagic and benthic food sources [58,89]. Biological transport processes such as the emergence of aquatic insects may also transfer Hg from aquatic food webs to adjacent terrestrial food webs [90]. Therefore, network structure, source analysis, and flux estimation capture different aspects of Hg transfer: network topology describes connectivity, source analysis compares the relative contributions of different pathways, and flux estimation further quantifies the actual amount of contaminant transferred. At present, studies that directly apply complex network approaches to the continuous transfer of Hg/MeHg remain relatively limited; therefore, this review does not treat general food-web surveys or source analyses uniformly as complex network analyses, but instead considers them important sources of evidence for defining nodes, pathways, and edge weights.

### 5.2. Bayesian Networks, Monte Carlo Simulation, and Probabilistic Risk Inference

When the research focus shifts from identifying the pathways along which Hg is transferred to estimating the likelihood of different risk states under varying monitoring evidence or management scenarios, BNs can be used for probabilistic analysis. A BN consists of a directed acyclic graph and corresponding probabilistic parameters, in which nodes represent model variables and directed arcs represent specified conditional dependencies; discrete BNs typically use conditional probability tables to describe the probability distribution of a child node under different combinations of parent-node states [19,20,85]. Network structure can be determined based on mechanistic knowledge, monitoring data, statistical relationships, or expert judgment; however, directed links primarily represent conditional dependencies, and their causal interpretation still requires support from relevant mechanistic evidence and study design. Existing Hg studies have demonstrated the use of Bayesian networks to integrate multiple sources of data. Jermilova et al. integrated water-column THg, Hg concentrations in edible fish tissues, and environmental and fish-related information from multiple publicly available datasets across the Mackenzie River Basin to develop a Bayesian-network relative-risk model for comparing changes in fish Hg, dietary intake, and the probabilities of different risk states under alternative management scenarios, together with sensitivity analysis [85]. Harris et al. and Landis et al. likewise incorporated Hg, other environmental stressors, ecological data, and information on human activities into Bayesian-network relative-risk models to support integrated assessment of different risk endpoints [91,92]. These studies demonstrate that Bayesian networks can integrate information from different sources and spatial or temporal scales within a common probabilistic framework and update the probabilities of target states in response to monitoring evidence or specified scenarios.

The parameterization of BNs should be determined according to variable types and data availability. Jermilova et al. used monitoring data and mixed-effects models to derive probabilistic parameters for selected nodes [85], whereas Bonotto et al. used continuous conditional distributions to characterize the relationship between sediment Hg concentrations and indicators of geological sources [93]. Discretization facilitates the construction of conditional probability tables, but different state definitions can affect probabilistic outcomes and increase the amount of data required for parameter estimation [19,94]. Bayesian methods can also be used for model parameter estimation; for example, Zhu et al. applied approximate Bayesian computation to estimate bioaccessibility parameters in a physiologically based toxicokinetic model of MeHg [95], which differs in purpose from the use of Bayesian networks for risk-state inference based on network structure.

Model evaluation is an important component of Bayesian network applications. Sensitivity analysis is used to identify variables that are strongly associated with model outputs, scenario analysis compares changes in risk states under different conditions, and predictive validation evaluates model performance using data not involved in parameter fitting [94,96,97]. Bonotto et al. compared predicted and observed values and residuals and used leave-one-out cross-validation to evaluate the corresponding regression relationships [93]. Jermilova et al. conducted parameterization, sensitivity analysis, and scenario analysis, although model validation remained constrained by limited monitoring data in some regions and insufficient consideration of seasonal variation [85]. Therefore, sensitivity analysis and scenario inference cannot substitute for predictive validation, and practical applications should evaluate model performance using holdout data, cross-validation, or independent temporal and spatial datasets, depending on the intended use of the model.

Unlike Bayesian networks, which update the probability of target states according to conditional dependencies, Monte Carlo simulation primarily propagates variability and uncertainty in input parameters to exposure or risk outcomes through repeated random sampling. Existing studies have used probability distributions for parameters such as fish MeHg concentrations, consumption amount, consumption frequency, and body weight in simulations, and have reported probability distributions, percentiles, and probabilities of exceeding specified thresholds for intake or risk metrics such as hazard quotient (HQ) [22,76,98]. Watanabe et al. used fish MeHg monitoring and consumption data to derive probability distributions of intake and examined how assumptions regarding input distributions affected estimates of upper-end exposure [22], whereas Krupa et al. represented parameters such as concentration, body weight, and consumption frequency using probability distributions and reported the 95th percentile of HQ [76]. Therefore, probabilistic risk assessment should specify not only the input distributions used but also whether the final outputs are reported as percentiles or probabilities of exceeding predefined thresholds.

Hg speciation and food processing can also influence the results of probabilistic exposure assessments. Lin et al. estimated MeHg concentrations from THg using MeHg/THg ratios and subsequently conducted Monte Carlo exposure analysis [82], whereas Charette et al. further incorporated parameters such as the proportion of MeHg, bioaccessibility, and cooking-induced weight loss into a probabilistic model [83]. These studies indicate that, in addition to population-specific consumption parameters, probabilistic exposure assessments should clearly specify the sources of parameters related to Hg speciation conversion, food processing, and bioaccessibility. Lackner et al. further integrated probabilistic exposure assessment with dose–response relationships and disability-adjusted life years to estimate the MeHg-related health burden [99], demonstrating that probabilistic analysis can be extended from dietary exposure to health outcomes, although this requires additional population health and dose–response data. Network analysis, BNs, and Monte Carlo simulation focus primarily on transfer structure, conditional probabilities, and parameter distributions, respectively, and differ in their research objectives, data requirements, and output formats, as summarized in Table 4.

### 5.3. Multi-Source Information Integration and Risk Early-Warning Framework for Fishery Products

**Figure 3:**
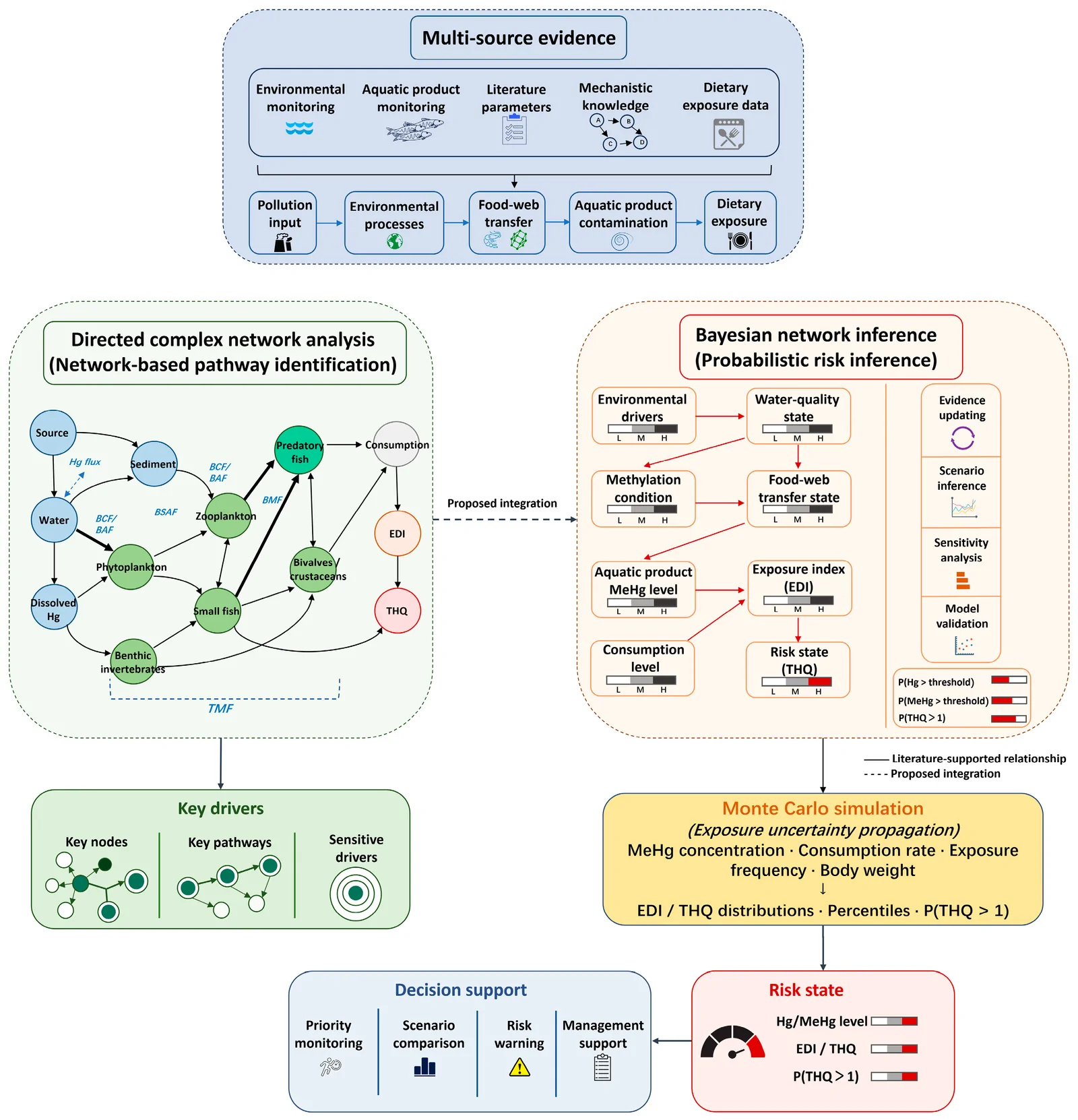
Conceptual framework for Hg/MeHg network-based pathway identification and probabilistic risk assessment. Multi-source evidence includes environmental monitoring, fishery-product monitoring, literature-derived parameters, mechanistic knowledge, and dietary exposure data. Network analysis is used to identify key nodes, major transfer pathways, and associated driving factors; BNs are used to integrate multiple sources of information and perform probabilistic inference of risk states, evidence updating, scenario analysis, and sensitivity analysis; and Monte Carlo simulation is used to propagate uncertainty in exposure parameters such as MeHg concentration, consumption amount, consumption frequency, and body weight, thereby generating probabilistic outputs for estimated daily intake (EDI), target hazard quotient (THQ), and P(THQ > 1). Solid lines represent methodological relationships supported by the existing literature, whereas dashed lines indicate the integrative relationships proposed in this review (proposed integration). Arrows indicate the direction of transfer, dependency, or information flow; blue, green, orange, yellow, and red elements denote evidence/decision support, network analysis/key drivers, Bayesian inference, Monte Carlo simulation, and risk states, respectively. The complete framework still requires further parameterization and independent validation.

Existing studies have separately demonstrated the applications of network analysis, BNs, and Monte Carlo simulation to Hg/MeHg pathway identification, probabilistic inference, and dietary exposure assessment [17,22,85]. Building on these studies, this review proposes a conceptual Network–BN–Monte Carlo framework to organize different types of evidence related to Hg/MeHg environmental transport, food-web transfer, contamination of fishery products, and dietary exposure. Network analysis is first used to identify priority nodes and candidate transfer pathways, and the resulting pathway information and monitoring evidence can then be incorporated into Bayesian networks for risk-state inference, while Monte Carlo simulation is used to propagate uncertainty in exposure parameters such as MeHg concentration, consumption amount, consumption frequency, and body weight. Harris et al. used Monte Carlo sampling to aggregate the probability distributions of different risk endpoints generated by a Bayesian network, providing an example of the combined application of Bayesian networks and Monte Carlo simulation [91]. Figure 3 summarizes the relationships among the three approaches within the framework proposed in this review.

The probability-based early warning discussed in this review refers to the use of monitoring data and probabilistic models to estimate the probability of a risk state or benchmark exceedance and to provide risk alerts based on changes in that probability as new evidence becomes available or under specified scenarios. Jermilova et al. compared the probabilities of risk states under different Hg deposition and fish consumption scenarios, illustrating one application of Bayesian-network scenario inference in risk management [85]. When model outputs are expressed as estimated daily intake (EDI), target hazard quotient (THQ), or P(THQ > 1), their interpretation should be based on the corresponding Hg species, exposure parameters, and health-based benchmarks; in particular, P(THQ > 1) represents only the probability that THQ exceeds 1 under the specified model and input conditions and does not represent the probability of disease occurrence. It should be noted that the complete integration of Network–BN–Monte Carlo methods remains a conceptual framework proposed in this review. When outputs from different methods are used in subsequent analyses, it is necessary to clarify whether the information being transferred represents pathways, concentrations, probability distributions, or model parameters, and to specify the corresponding data sources and uncertainties. If the framework is further applied to operational risk early warning, parameterization and independent validation using real monitoring data will be required, together with the specification of data-updating procedures, warning thresholds, and response rules according to monitoring conditions and management objectives.

## 6. Conclusions and Perspectives

The transfer of Hg/MeHg from aquatic environments to fishery products and subsequent human dietary exposure involves multiple interconnected processes, including environmental transport and transformation, food-web transfer, accumulation in edible tissues, and human consumption. Hg speciation and bioavailability in the water column, particulate matter, and sediments are influenced by redox conditions, organic matter, eutrophication, algal blooms, and seasonal variation, which in turn affect MeHg production and its pathways of entry into aquatic food webs. Once MeHg enters the food web, its accumulation in aquatic organisms is further influenced by species, trophic level, feeding habits, habitat, and individual size, while eventual human exposure also depends on MeHg concentrations in edible tissues and population-specific consumption patterns. Therefore, MeHg risk associated with fishery products cannot be assessed solely on the basis of a single environmental medium or concentration metric, but should instead be evaluated along the continuous pathway linking environmental media, food webs, fishery products, and human exposure.

At present, substantial mechanistic, monitoring, and exposure evidence has accumulated for different stages of the Hg/MeHg transfer process, but sampling locations, sampling periods, species, tissue types, and Hg species are not always directly aligned, and observations that continuously link environmental media, food webs, fishery products, and human exposure within the same aquatic system and over comparable time scales remain limited [3,33,58,76]. In addition, data sources differ among direct MeHg measurements, estimates derived from THg using conversion ratios, and model predictions [13,16,32,83]. Structural position within a network does not directly represent the actual magnitude of contaminant transfer, while the results of Bayesian networks and Monte Carlo simulations are influenced by factors such as network structure, probabilistic parameters, state discretization, and input distributions [17,22,85,94,97]. Therefore, when data and model outputs from different sources are compared or integrated, Hg species, sample type, data acquisition method, and the basis for parameterization should be clearly specified.

Future research should further strengthen coordinated monitoring with matched spatial and temporal coverage by simultaneously collecting Hg/MeHg data from the water column, particulate matter, sediments, planktonic and benthic organisms, and major edible fishery products in representative lakes, reservoirs, or aquaculture waters, while also recording key parameters related to food-web transfer and dietary exposure [3,58,61,63,66]. Food-web studies can further combine information on Hg/MeHg concentrations, dietary proportions, consumption rates, and source apportionment with the identification of nodes and pathways to estimate the actual transfer contributions of major trophic links [17,66,89]. Probabilistic models should also clearly specify whether each parameter is derived from direct measurements, the literature, or model assumptions, and their performance should be evaluated using independent data from subsequent years or other regions [19,22,94,97].

Based on the above analysis, this review proposes an integrated Network–BN–Monte Carlo framework to place Hg/MeHg environmental processes, food-web transfer, contamination of fishery products, and dietary exposure within a unified analytical framework. Network analysis is used to identify key nodes and major transfer pathways, BNs are used to compare changes in the probabilities of risk states under different monitoring evidence or management scenarios, and Monte Carlo simulation is used to propagate uncertainty in dietary exposure parameters. At present, this framework is more appropriately regarded as a conceptual framework for organizing different types of evidence and analytical approaches. For further application to operational risk early warning, parameterization and independent validation using real monitoring data are still required, together with the definition of data-updating procedures, warning thresholds, and response rules according to specific management needs.

## Figures and Tables

**Table 1:** Representative Regulatory Limits and Health-Based Guidance Values for Hg/MeHg in Fishery Products.

Issuing Authority/Region	Document or Assessment Basis	Type	Hg Species	Applicable Products and Representative Values
Codex Alimentarius Commission	General Standard for Contaminants and Toxins in Food and Feed, CXS 193-1995 [25]	Maximum Level	MeHg	Alfonsino, 1.5 mg/kg; marlin, 1.7 mg/kg; orange roughy, 0.8 mg/kg; pink cusk-eel, 1.0 mg/kg; shark, 1.6 mg/kg; and tuna, 1.2 mg/kg; applicable to the fresh/frozen commodity portions specified in the standard; see the sampling plan in Appendix IX of CXS 193-1995 for details.
European Commission	Commission Regulation (EU) 2023/915 [26]	Maximum Level	Hg (regulated as total Hg)	Maximum levels of 0.30, 0.50, or 1.0 mg/kg wet weight are established according to the category and species of fishery products.
United States Food and Drug Administration	Compliance Policy Guide Section 540.600 [27]	Action Level	MeHg (expressed as Hg)	The action level for MeHg in the edible portion, expressed as Hg, is 1 ppm (=1 mg/kg); this value is used as an enforcement action level.
Joint FAO/WHO Expert Committee on Food Additives (JECFA)	MeHg toxicological evaluation [11]	Provisional Tolerable Weekly Intake	MeHg	1.6 μg/(kg bw·week).
European Food Safety Authority (EFSA)	Scientific Opinion on the Risk for Public Health Related to the Presence of Mercury and Methylmercury in Food [29]	Tolerable Weekly Intake	MeHg (expressed as Hg)	1.3 μg/(kg bw·week)
United States Environmental Protection Agency (U.S. EPA)	Integrated Risk Information System Assessment for Methylmercury [12]	Reference Dose	MeHg	0.1 μg/(kg bw·day)
China	National Food Safety Standard—Maximum Levels of Contaminants in Foods (GB 2762—2025) [28]	Maximum Level for Food Contaminants	MeHg (expressed as Hg)	Aquatic animals and their products (excluding carnivorous fish), 0.5 mg/kg; general carnivorous fish, 1.0 mg/kg; tuna, 1.2 mg/kg; alfonsino, 1.5 mg/kg; marlin, 1.7 mg/kg; shark, 1.6 mg/kg.

Note: Food maximum levels and action levels are primarily used for regulatory compliance, whereas human dietary exposure is assessed using the provisional tolerable weekly intake (PTWI), tolerable weekly intake (TWI), and reference dose (RfD). These indicators differ in their applicable populations or products, Hg species, and time scales and therefore should not be directly compared numerically. The Codex reference is based on the 2025 revised text, while the EU reference is based on the consolidated text dated 19 August 2026. GB 2762—2025 entered into force on 2 September 2026 and generally applies to the edible portion; for dried products, dehydration and concentration factors should be considered in accordance with the provisions of the standard. bw, body weight; Hg, mercury; MeHg, methylmercury.

**Table 2:** Representative Studies on Hg/MeHg Transport and Transformation, Environmental Regulation, and Food-Web Transfer.

Reference	Study Type, System, and Key Conditions	Main Hg/MeHg Findings and Significance
Microbial methylation and environmental conditions		
Parks et al. [6]	Culture experiments: two model Hg-methylating microorganisms; *hgcA*/*hgcB* gene deletion and complementation.	*hgcA* and *hgcB* are essential genes for Hg methylation in these two model microorganisms. Detection of *hgcAB* in environmental samples indicates methylation potential but does not directly represent actual MeHg production.
Capo et al. [8]	Field sampling and tracer incubation: stratified water columns at two sites in the Baltic Sea; analysis of genes, transcripts, Hg species, and isotope-tracer incubations.	Transcript abundance should be interpreted together with information such as dissolved Hg(II) sulfide species to explain variations in Hg methylation. In some transition layers, methylation rates remained low despite the detection of *hgc* transcripts.
Fuhrmann et al. [42]	Microcosm experiment: sediment–water interface from a sulfate-rich, hypereutrophic reservoir.	Different redox conditions and microbial inhibition treatments affected net MeHg accumulation in the aqueous phase, with the direction of change depending on the specific experimental conditions.
Millard et al. [47]	Field sampling, tracer incubation, and model analysis: a contaminated tributary of Nacimiento Reservoir, USA, sampled in winter and summer.	During summer stratification and anoxia, water-column methylation may represent an important source of MeHg in bottom waters, while the relative contributions of the water column and sediments vary seasonally.
Environmental drivers related to eutrophication, algal blooms, and CO_2_		
Lei et al. [49]	Sediment microcosm experiments using samples from 10 lakes in China; 1000 mg/L algal biomass was added followed by 21 days of anaerobic dark incubation.	Compared with controls without algal addition, sediment MeHg concentrations increased by 54–514%, while Archaea-*hgcA* abundance increased by 51–397%. These results indicate that, under the experimental conditions used, algal biomass inputs can promote MeHg formation in sediments.
Balzer et al. [50]	Seasonal vertical observations in Lake Ölper, Germany, from April to November 2019, with particular emphasis on Hg speciation in suspended particles > 25 μm.	The MeHg/THg ratio in suspended particles reached a maximum of approximately 26% at the oxic–suboxic boundary, while the period of peak algal biomass did not coincide with the months of elevated MeHg concentrations. The value of 26% represents the proportion of MeHg in THg rather than an increase in MeHg concentration.
Li et al. [51]	Spatial survey of 17 sampling sites in Lake Taihu, comparing Hg accumulation characteristics in phytoplankton and zooplankton across areas with different algal densities.	Phytoplankton THg BCFs, zooplankton THg BAFs, and planktonic MeHg/THg ratios were generally lower in areas with high algal density. The authors considered algal bloom dilution insufficient to explain this pattern. This study reflects only spatial differences among sampling sites.
Lei et al. [53]	Lake-water microcosm experiments using samples from three eutrophic shallow lakes; Hg(II) was added, followed by anaerobic dark incubation, with treatments comparing the presence and absence of algal-derived organic matter.	Dissolved MeHg levels were higher after the addition of algal-derived organic matter than in the corresponding controls without algal addition. This result reflects the effect of algal-derived organic matter on MeHg formation under Hg(II)-spiked incubation conditions.
Wu et al. [56]	Rice–fish–snail wetland microcosm experiment comparing 600 ppm CO_2_ with ambient CO_2_ conditions.	Elevated CO_2_ increased MeHg levels in the water and in some organisms. This result reflects the association between elevated CO_2_ and changes in MeHg accumulation within this integrated wetland system.
Lei et al. [57]	Hg-spiked microcosm experiments using water samples from 45 freshwater lakes, with a 7-day incubation comparing 1000 ppm and 420 ppm CO_2_ conditions.	Compared with 420 ppm, net MeHg production under 1000 ppm CO_2_ decreased by 14–96%, with an average reduction of 63%. These results indicate that, under the experimental conditions used, elevated CO_2_ can reduce net MeHg production.
Food-web accumulation and trophic transfer		
Chen et al. [3]	Field survey of 10 estuaries in the northeastern United States, analyzing MeHg in environmental media and in fish, crabs, mussels, and worms; whole-body tissues were analyzed for small fish.	MeHg in particulate matter was more closely associated with MeHg in small fish, whereas sediment MeHg showed a stronger relationship with MeHg in worms, indicating that MeHg accumulation in different biological groups may be linked to distinct environmental media and food sources.
Wang et al. [9]	Field food-web survey in Lake Taihu, with measurements of THg and MeHg combined with stable-isotope analysis.	Both THg and MeHg concentrations increased with trophic level, while stable-isotope results indicated that pelagic food sources represented an important energy source for the food web in the study area.
Sinclair et al. [10]	Meta-analysis including 58 studies and 365 freshwater sites, with comparisons across taxonomic groups, feeding guilds, and methods for estimating trophic level.	The magnitude of MeHg biomagnification varied among taxonomic groups, feeding guilds, and trophic-level estimation methods, indicating that trophic magnification results are influenced by food-web composition and analytical approach.
Jonsson et al. [62]	Estuarine mesocosm Hg isotope-tracer experiment comparing water-column and different sediment sources, with pooled dry-weight samples of four groups of benthic invertebrates analyzed.	Hg location, chemical form, and food source all influenced MeHg accumulation in benthic fauna, with source contributions differing among biological groups.

Note: The findings summarized in the table correspond to the specific experimental or observational conditions of each study and should not be interpreted as universal patterns across all lakes or fishery products. Percentages are reported according to the indicators and denominators defined in the original studies. Relative changes in concentration are not equivalent to methylation rates, and MeHg/THg represents the proportion of MeHg in THg rather than the percentage increase in MeHg concentration. BAF, bioaccumulation factor; BCF, bioconcentration factor; Hg, mercury; MeHg, methylmercury; THg, total mercury.

**Table 3:** Directly Measured MeHg Levels and Analytical Methods for Edible Fish Reported in Recent Studies.

Reference and Region	Publication/ Sampling Year	Study Subjects and Samples	MeHg Concentration (mg/kg, Wet Weight)	Analytical Method	Data Characteristics
Storelli et al. [33], Italy	2022/2020	Fifteen elasmobranch (shark and ray) species; edible muscle tissue; pooled samples prepared from 20–52 individual fish per species.	Species-specific mean concentrations: 0.41–1.10	Extraction and derivatization followed by GC-MS; THg determined by acid digestion and AAS.	MeHg concentrations varied among species.
Donald and Bernhard [72], Norway	2025/2024.	Fillets from four farmed fish species; each pooled sample consisted of three individual fish.	Species-specific median concentrations: approximately 0.015–0.068; maximum concentrations: 0.028–0.075	GC-ICP-MS	Monitoring data for farmed fish, with substantial variation in sample size among species.
Hernández-Mendiola et al. [31], Mexico.	2026/2018	Muscle tissue from three market fish species; two samples per species were subjected to Hg speciation analysis.	Species-specific mean concentrations: 0.18–0.45	HPLC-ICP-MS	The sample size was small, and Hg speciation was determined directly.

Note: All concentrations listed in the table are wet-weight values reported in the original studies and are expressed in mg/kg. All MeHg concentrations were determined directly by speciation analysis and do not include values estimated from THg using conversion ratios. Data from Storelli et al. [33] are reported as mean ± SD for pooled samples by species; data from Donald and Bernhard [72] are reported as median/maximum values; and data from Hernández-Mendiola et al. [31] are reported as mean ± SD for two muscle samples per fish species. AAS, atomic absorption spectrometry; GC-MS, gas chromatography–mass spectrometry; GC-ICP-MS, gas chromatography–inductively coupled plasma mass spectrometry; HPLC-ICP-MS, high-performance liquid chromatography–inductively coupled plasma mass spectrometry.

**Table 4:** Comparison of Network Analysis, Bayesian Networks, and Monte Carlo Simulation.

Method	Main Purpose	Main Data or Inputs	Representation of Relationships or Uncertainty	Main Outputs	Main Limitations and Role in the Framework Proposed in This Review
Network analysis	Describe nodes and their connections and identify key nodes and major transfer pathways	Nodes, feeding relationships, contaminant concentrations, dietary composition, transfer fluxes, or other edge-weight data	Edges represent relationships such as feeding interactions, associations, or material transfer, with their specific meanings determined by the study design	Network structural metrics, key nodes, major pathways, and, when edge weights are available, pathway contributions	Results are influenced by network boundaries, definitions of nodes and edges, and weighting schemes; structural importance cannot substitute for actual transfer magnitude. In the framework proposed in this review, network analysis is mainly used to identify Hg/MeHg transmission structures and priority pathways.
Bayesian network (BN)	Describe conditional dependencies among variables and update the probabilities of target states based on new evidence or specified scenarios	Network structure, monitoring data, conditional probabilities or continuous conditional distributions, and information from the literature, existing models, or expert knowledge	Conditional dependencies among variables are represented by directed arcs and conditional probabilities	Posterior probabilities, probabilities of risk states, scenario inference, and sensitivity analysis results	Results are influenced by network structure, parameter quality, state discretization, and the extent of model validation; directed arcs cannot automatically be interpreted as causal relationships. In the framework proposed in this review, BNs are used to integrate information from multiple sources and compare the probabilities of risk states under different evidence or scenarios.
Monte Carlo simulation	Propagate input parameter distributions to exposure or risk outcomes and characterize the probability distributions of the resulting outputs	Probability distributions of MeHg concentration, consumption amount, body weight, consumption frequency, and other parameters	Variability and uncertainty in parameters are represented through repeated random sampling	Probability distributions, percentiles, and probabilities of exceeding specified thresholds for exposure or risk metrics	Results depend on the specified input distributions, correlations among parameters, and data representativeness, while the simulation itself does not establish causal mechanisms. In the framework proposed in this review, Monte Carlo simulation is mainly used to propagate uncertainty in dietary exposure parameters and derive probabilistic estimates of EDI, THQ, and related metrics.

Note: The three methods can be applied independently according to the research objectives and data availability, or combined when variable relationships, parameter meanings, and data sources are clearly defined.

## Data Availability

No new data were created or analyzed in this study. Data sharing is not applicable to this article.

## Abbreviations

The following abbreviations are used in this manuscript:

BAF	Bioaccumulation factor
BCF	Bioconcentration factor
BMF	Biomagnification factor
BN	Bayesian network
BSAF	Biota-sediment accumulation factor
DO	Dissolved oxygen
DOC	Dissolved organic carbon
DOM	Dissolved organic matter
EDI	Estimated daily intake
EPA	U.S. Environmental Protection Agency
Hg	Mercury
JECFA	Joint FAO/WHO Expert Committee on Food Additives
MeHg	Methylmercury
ORP	Oxidation-reduction potential
RASFF	Rapid Alert System for Food and Feed
RfD	Reference dose
THg	Total mercury
THQ	Target hazard quotient
TMF	Trophic magnification factor
TN	Total nitrogen
TP	Total phosphorus
